# Prophylactic or therapeutic administration of *Holarrhena floribunda* hydro ethanol extract suppresses complete Freund’s adjuvant-induced arthritis in Sprague-Dawley rats

**DOI:** 10.1186/s12950-022-00301-2

**Published:** 2022-03-05

**Authors:** Stephen Antwi, Daniel Oduro-Mensah, Jerry Asiedu-Larbi, Ebenezer Oduro-Mensah, Olga Quasie, Clara Lewis, David Darko-Obiri, Augustine Ocloo, Laud Kenneth Okine

**Affiliations:** 1 Department of Pharmacology/Toxicology, Centre for Plant Medicine Research, P. O. Box 73, Mampong, Akuapem Ghana; 2 Department of Pharmacology, Faculty of Pharmacy and Pharmaceutical Sciences, College of Health Sciences, Kwame Nkrumah University of Science and Technology, Kumasi, Ghana; 3 Department of Biochemistry, Cell and Molecular Biology, College of Basic and Applied Sciences, University of Ghana, P. O. Box LG 54, Accra, Ghana; 4 West African Centre for Cell Biology of Infectious Pathogens, College of Basic and Applied Sciences, University of Ghana, Accra, Ghana; 5 Ga-East Municipal Hospital, Kwabenya, Accra, Ghana; 6 Clinical Research Department, Centre for Plant Medicine Research, P. O. Box 73, Mampong, Akuapem Ghana

**Keywords:** Arthritis, Cytokines, *Holarrhena floribunda*, Immunomodulation, Inflammation

## Abstract

**Background:**

A hydro ethanol extract of the stem bark of *Holarrhena floribunda* (HFE) has been shown to be effective in the management of acute inflammation. This study was to evaluate usefulness of the extract for the management of chronic inflammation in a murine model.

**Methods:**

Arthritis was induced in Sprague-Dawley rats using Complete Freund’s Adjuvant. Anti-arthritic effect of the extract was evaluated in prophylactic and therapeutic treatment models at doses of 50, 200 and 500 mg/kg. Parameters assessed included oedema, serology of inflammatory response, bone tissue histology and haematology. Data were analysed by ANOVA and Tukey’s multiple comparisons post hoc test.

**Results:**

HFE at 50–500 mg/kg dose-dependently [*P* ≥ 0.0354 (prophylactic) and *P* ≥ 0.0001 (therapeutic) inhibited swelling of the injected paw upon prophylactic [≤ 81.26% (*P* < 0.0001) or therapeutic [≤ 67.92% (*P* < 0.01) administration — and prevented spread of arthritis to the contralateral paw. The inflammation alleviation activity was further demonstrated by decrease in arthritis score, radiologic score and erythrocyte sedimentation rate. HFE at all doses significantly reduced serum interleukin (IL)-1α (*P* < 0.0197), and 500 mg/kg HFE reduced IL-6 (*P* = 0.0032). In contrast, serum concentrations of IL-10, protein kinase A and cyclic adenosine monophosphate were enhanced (*P* ≤ 0.0436). HFE consistently showed better prophylactic than therapeutic activity.

**Conclusion:**

HFE strongly suppressed Complete Freund’s Adjuvant-induced arthritis and modulated regulators of inflammation, including IL-1α, − 6 and − 10. Taken together, the data suggest that HFE has potential for use as an agent for modulation of the inflammatory response.

**Supplementary Information:**

The online version contains supplementary material available at 10.1186/s12950-022-00301-2.

## Introduction

Currently, chronic diseases are the major cause of mortality worldwide (WHO, 2019). Inflammation has been shown to have a strong and consistent relationship with several chronic disease conditions [1–5]. In addition to reduced quality of life due to disability, the economic burden of chronic inflammatory diseases is clearly demonstrable [6–9]. Long-term treatment options, however, are still not optimal. With respect to rheumatoid arthritis, for example, oral anti-rheumatics have demonstrated limited efficacy and significant toxicity in humans [10–15]. As inflammation-related acute or chronic conditions emerge and//or persist, it is important to continually prospect for treatment alternatives that may have relatively acceptable or no undesirable side effects. Such agents should be more suitable for safe prophylactic use or long-term management of acute and chronic inflammatory diseases, as well as inflammation-related metabolic diseases.

*Holarrhena floribunda* (G.Don) T. Durand & Schinz is a medium-sized tree of the family *Apocynaceae*. In many parts of West Africa, extracts of the leaves, bark and roots have popular use for management or treatment of conditions including hypertension, diabetes, diarrhoea, malaria, venereal diseases, kidney pain and snake bite [16–21]. The stem bark has use in Ghanaian folk medicine for the treatment of inflammation-related conditions such as diabetes and hypertension. Using murine models of acute inflammation, data from our laboratory show that the hydro ethanol extract of *H. floribunda* stem bark has both prophylactic and therapeutic anti-inflammatory and antihistaminic effects [22], showing [1] protection from anaphylaxis by suppressing sepsis or systemic shock due to lipopolysaccharide and compound 48/80, respectively, and [2] inhibitory effect on paw oedema due to phlogistic agents including carrageenan, histamine, serotonin and prostaglandin E_2_. The mean lethal dose in Sprague Dawley rats and Imprint Control Region mice was found to be greater than 5000 mg/kg [22]. This study was designed to evaluate the in vivo anti-inflammatory activity of *H. floribunda* stem bark hydro ethanol extract (HFE) in a chronic inflammation model, and to identify mediators of the observed biological activity. Prophylactic and therapeutic anti-inflammatory activities of HFE were assessed.

## Results

### Maximal and Total Oedema

**Fig. 1:**
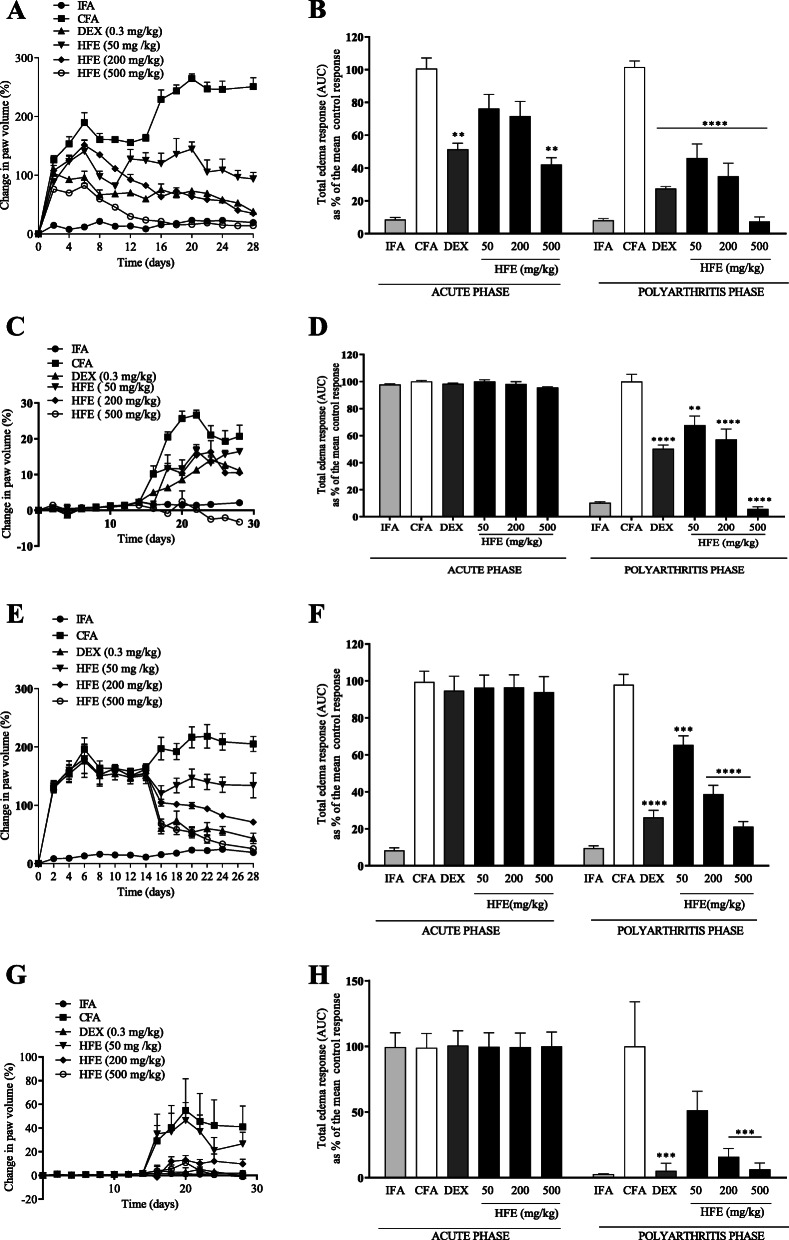
Inhibition of adjuvant-induced arthritis in Sprague-Dawley rats. Rats were injected sub plantar with CFA or IFA into the right hind paw. Paw volumes were monitored in both the injected [A, E and contralateral [C, G paw for up to 28 days. Total oedema was calculated as area under the time course curves, AUC [B, D, F, and G. The vehicle, dexamethasone (0.3 mg/kg) or HFE was administered 1 h before induction of arthritis and daily for 28 days in the prophylactic model [A–D or administered from day 14 after induction and continued daily for 28 days in the therapeutic model [E–H. The plotted values are mean ± SEM for *n* ≥ 5. * (*P* ≤ 0.05), ** (*P* ≤ 0.01), *** (*P* ≤ 0.001), **** (*P* ≤ 0.0001) values significantly different from CFA arthritic control group

Paw oedema as a measure of arthritis development in the arthritis control group showed that arthritis progression was in two phases: an acute inflammatory stage between days 2–8, followed by further increase which indicated progression to chronic polyarthritis from day 14 and peaking on day 20 (Fig. 1a & e). In the injected paw, maximal oedema for the prophylactic and therapeutic treatment models were 261.57% (Fig. 1a) and 219.35% (Fig. 1e) of baseline paw volume, respectively. In the contralateral paw, maximal oedema values were 26.78% of baseline for the prophylactic model (Fig. 1c) and 54.98% for the therapeutic model (Fig. 1g).

HFE exhibited anti-arthritic activity (Fig. 1b & f) and inhibited spread of arthritis to the contralateral paw (Fig. 1d & h) in the prophylactic treatment model. In the acute inflammation phase, 500 mg/kg HFE reduced total oedema by 56.59%, similar (*P* = 0.9632) to oedema reduction by dexamethasone (48.77%) (Fig. 1b). HFE reduced oedema in the polyarthritis phase by ≥53.94% (Fig. 1 B), with reduction at 500 mg/kg (92.56%) being higher (*P* = 0.3080) than reduction by dexamethasone (77.99%). Again, HFE reduced contralateral oedema due to polyarthritis by ≥32.49% (Fig. 1d), with reduction by 500 mg/kg HFE (94.15%) significantly higher (*P* < 0.0001) than reduction by dexamethasone (49.73%). There were no differences (*P* > 0.9993) in oedema between the treatment groups during the acute inflammation phase of the therapeutic model (Fig. 1f & h). In the polyarthritis phases, HFE inhibited oedema by ≥34.69% for the injected paw and ≥ 48.79 for the contralateral paw (Fig. 1f & h). Inhibition by 200 or 500 mg/kg HFE was not different (*P* ≥ 0.6142) from inhibition by dexamethasone for both.

### Arthritis score

#### Photograph scoring

**Fig. 2:**
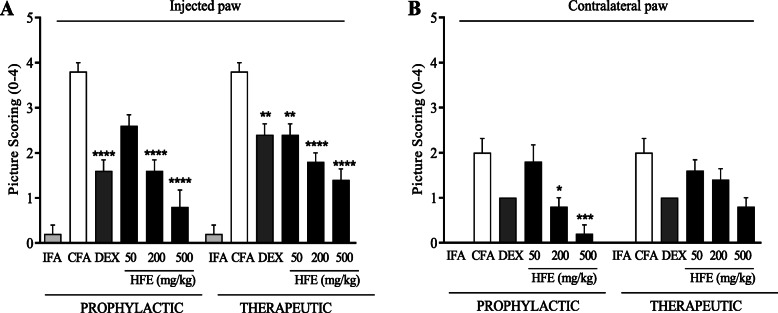
Arthritis score from photographs of arthritic Sprague-Dawley rats. The rats were injected sub plantar in the right hind paw with IFA or CFA. Vehicle, dexamethasone (0.3 mg/kg) or HFE treatment was administered 1 h before arthritis induction and daily thereafter until day 28 in the prophylactic model (left panel). In the therapeutic model, treatment was from day 14–28 (right panel). Arthritis scores were assigned on a scale of 0–4. The plots show scores as mean ± S.E.M of n ≥ 5. * (*P* ≤ 0.05), ** (*P* ≤ 0.01), *** (*P* ≤ 0.001), *** (*P* ≤ 0.0001) values significantly different from CFA arthritic control group

**Plate 1:**
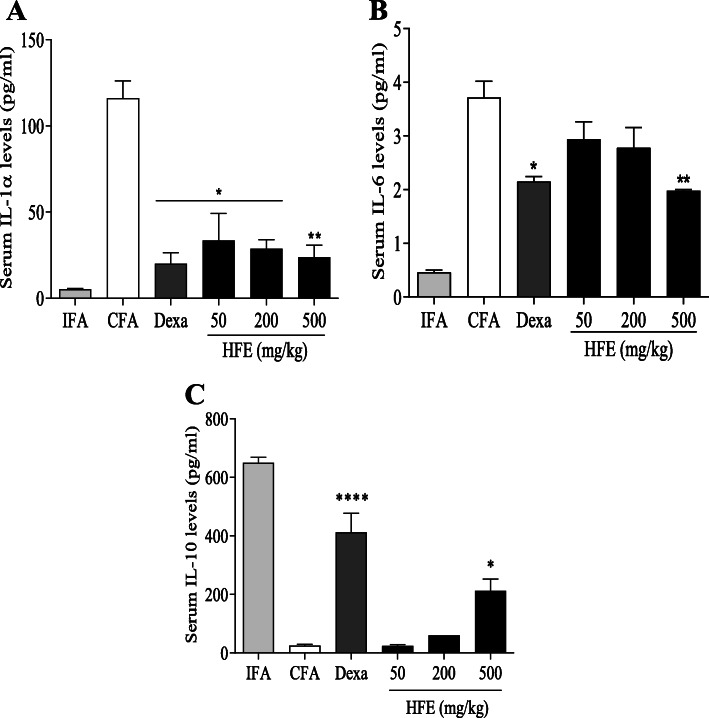
Effect of treatments on adjuvant-induced arthritis in Sprague-Dawley rats. The rats were injected sub plantar with IFA or CFA in the right hind paw. Vehicle, dexamethasone (0.3 mg/kg) or HFE treatment was administered 1 h before arthritis induction and daily thereafter until day 28 in the prophylactic model. In the therapeutic model, treatment was from day 14–28. (A) IFA/non-arthritic control; (B) CFA/arthritic control; (C) Dexamethasone (representative); (D–F) 50–500 mg/kg HFE prophylactic; (G–I) 50–500 mg/kg HFE therapeutic

The IFA control group had the lowest arthritis score for the injected (Fig. 2a) and contralateral (Fig. 2b) paws, with no visible signs of oedema or erythema on day 28 (Plate 1 A). The untreated CFA control group scored highest, with photographs showing severe erythema, lesions and/or swelling of soft tissue in both paws for the prophylactic and therapeutic models (Plate 1 B). Dexamethasone-treated rats showed moderate levels of erythema, soft tissue swelling and lesions in both the injected and contralateral limbs (Plate. 1 C). In all cases, administration of HFE reduced the arthritis scores and inhibited the development of erythema, lesion and swelling (Plate 1 D–I) in both the injected and contralateral paws. Efficacy of HFE appeared to increase when administered prophylactically (Fig. 2), whereas dexamethasone did not show any such indication.

#### Radiography

**Plate 2:**
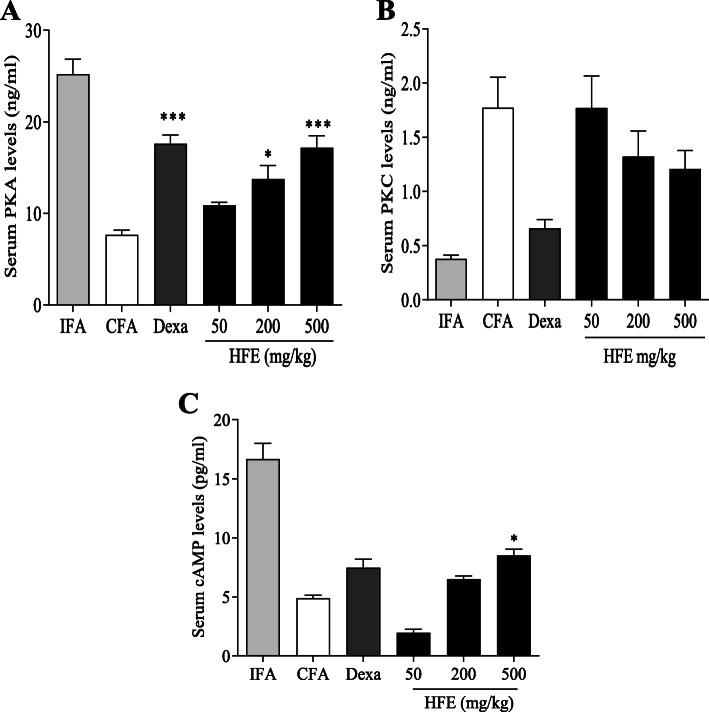
Radiographs of control and arthritic Sprague-Dawley rats. The rats were injected sub plantar with IFA or CFA in the right hind paw. Vehicle, dexamethasone (0.3 mg/kg) or HFE treatment was administered 1 h before arthritis induction and daily thereafter until day 28 in the prophylactic model. In the therapeutic model, treatment was from day 14–28. (A) IFA/non-arthritic control; (B) CFA/arthritic control; (C) Dexamethasone; (D–F) 50–500 mg/kg HFE prophylactic; (G–I) 50–500 mg/kg HFE therapeutic

IFA control rats showed no indication of joint damage or osteolysis of bone in the injected or contralateral hind paws (Plate 2 A, Table 2). In the untreated CFA control group, there was evidence of severe periarticular soft tissue swelling in both the injected and contralateral hind limbs (Plate 2 B). Osteolysis of tarsal and metatarsal bones, as evidenced by reduced bone density and demineralization of the bones, was observed in the untreated CFA group. In addition, there were signs of inflammation at the metatarsal-phalangeal joint and the regions in-between the bones of the phalanges and the metatarsals. Erosion of the phalangeal bone was observed, with moderate affectation of the distal tibia and joint deformation. The untreated CFA group radiographs recorded the highest arthritis blind scores (Table 1). Administration of dexamethasone resulted in reduced soft tissue swelling, osteolysis of tarsal and metatarsals bone, moderate affectation of distal tibia, reduced joint deformation (Plate 2 C) and reduced arthritic score (Table 1). Prophylactic (Plates 2 D–F) or therapeutic (Plates 2 G–I) HFE administration reduced peri-articular soft tissue swelling, radiographic joint lesion, bone demineralization and erosion, and joint deformation. The HFE treated rats received lower scores compared to the CFA control group (Table 1), and scores for 500 mg/kg HFE were at least equal to dexamethasone scores.

**Table 1:** Radiologic score of adjuvant-induced arthritic rats

	Peri-articular swelling	Osteolysis	Joint destruction
IFA	0	0	0
Control			
CFA	3	3	3
Dexamethasone	1	1	2
Prophylactic treatment			
HFE (50 mg/kg)	3	2	2
HFE (200 mg/kg)	2	2	2
HFE (500 mg/kg)	1	1	1
Therapeutic treatment			
HFE (50 mg/kg)	3	2	2
HFE (200 mg/kg)	2	2	2
HFE (500 mg/kg)	1	1	1

Sprague-Dawley rats were injected sub plantar with 0.1 ml of IFA or CFA into the right hind paw. The drug vehicle, dexamethasone (0.3 mg/kg) and HFE (50, 200, 500 mg/kg) were administered orally 1 h before arthritis induction and thereafter until the 28th day in the prophylactic approach and started on the 14th day after the induction of arthritis and daily till the 28th day in the therapeutic approach. Radiographs were taken. The extent of peri-articular swelling, oesteolysis and joint damage were blindly scored on a scale of 0–3, where 0: no damage; 1: mild; 2: moderate; and 3: severe. Values shown are mean of n ≥ 5

#### Histology

**Plate 3:**
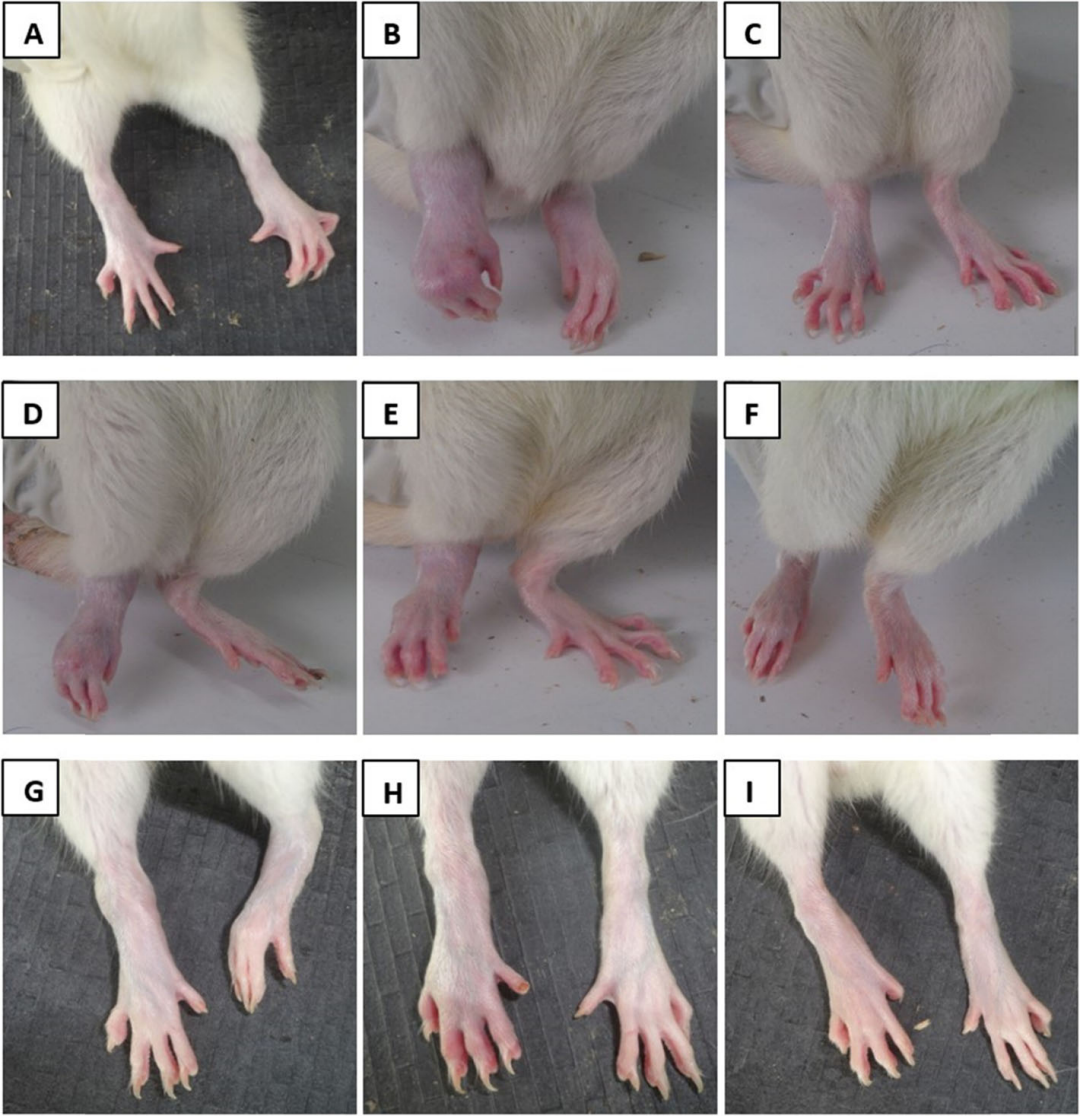
Photomicrographs of paw sections of control and arthritic Sprague-Dawley rats. The paw sections were stained with hematoxylin-eosin. (A) Control IFA rats; (B) arthritis (CFA) control rats; (C) 0.3 mg/kg dexamethasone-treated rats; (D–F) 50–500 mg/kg HFE prophylactic; (G–I) 50–500 mg/kg HFE therapeutic. Magnification: × 100

IFA control rats had intact bone structure with no visible necrotising granulomatous inflammation and mononuclear cell infiltration (Plate 3 A). The CFA arthritic control group showed necrotising granulomatous inflammation of the synovial membrane and bone erosion (Plate 3 B). There was severe mononuclear infiltration with mostly lymphocytes and multinucleated giant cells, as well as vascular proliferation and presence of macrophages. Arthritic changes observed in the CFA control group were ameliorated on treatment with dexamethasone or HFE (Plate 3, Table 2).

**Table 2:** Histology scores on micrographs of paw sections from control and adjuvant-induced arthritic Sprague-Dawley rats

	Histology score	
	Prophylactic	Therapeutic
IFA	0	0
CFA	3	3
Dexamethasone (0.3 mg/kg)	1	1
HFE (50 mg/kg)	2	2
HFE (200 mg/kg)	2	2
HFE (500 mg/kg)	1	1

Sprague-Dawley rats were injected sub plantar with 0.1 ml of IFA or CFA into the right hind

paw. The drug vehicle, dexamethasone (0.3 mg/kg) and HFE (50, 200, 500 mg/kg) were administered orally 1 h before arthritis induction and thereafter until the 28th day in the prophylactic approach and started on the 14th day after the induction of arthritis and daily till the 28th day in the therapeutic approach. Bone tissue of the right hind limbs were sectioned, stained with haematoxylin-eosin and micrographs of the tissue were scored on a scale of 0–3: (0) absence of inflammation indicators [1]; mild inflammation [2]; moderate inflammation [3]; inflammation. Values shown are mean of *n* ≥ 5

### Haematology

Induction of arthritis increased (*P* ≤ 0.0004) white blood cell (WBC) count and erythrocyte sedimentation rate (ESR), whilst decreasing haematocrit (*P* < 0.0001) in the CFA control relative to the IFA control (Table 3). Prophylactic HFE administration at 200 or 500 mg/kg had a modulatory effect, reducing WBC (*P* ≤ 0.0144) and ESR (*P* < 0.0001) — and increasing haematocrit (*P* < 0.0011) compared to the CFA control. Therapeutic HFE reduced ESR (*P* ≤ 0.0001) and increased haematocrit (*P* ≤ 0.0185). Unlike therapeutic dexamethasone, prophylactic dexamethasone resulted in an indication of leukopenia (*P* < 0.0001).

**Table 3:** Effect of treatment on haematological profile of adjuvant-induced arthritic rats

		WBC (×10^3^/μL)	RBC (× 10^6^/μL)	HGB (g dL^−1^)	HCT (%)	ESR (mm/h)
Control	IFA	13.58 ± 1.25	8.18 ± 0.14	15.15 ± 0.22	47.15 ± 0.79	0.40 ± 0.24
	CFA untreated	18.94 ± 0.85	6.83 ± 0.395	12.96 ± 0.25	40.26 ± 1.33	10.00 ± 1.87
Prophylactic	Dexamethasone	7.00 ± 0.56¥	8.31 ± 0.18	14.74 ± 0.25	45.34 ± 0.97#	2.20 ± 0.94#
	HFE (50 mg/kg)	16.65 ± 1.8	7.81 ± 0.14	13.85 ± 0.18	42.38 ± 0.86	6.50 ± 1.19
	HFE (200 mg/kg)	14.68 ± 0.2*	8.08 ± 0.22	14.80 ± 0.37	45.35 ± 0.87**	3.40 ± 0.93¥
	HFE (500 mg/kg)	13.97 ± 1.32**	8.29 ± 0.26	14.96 ± 0.11	46.24 ± 0.27¥	1.60 ± 0.68¥
Therapeutic	Dexamethasone	15.50 ± 1.61	8.76 ± 0.22	15.48 ± 0.42	48.33 ± 1.62	2.10 ± 0.10¥
	HFE (50 mg/kg)	18.78 ± 0.75	7.86 ± 0.39	14.83 ± 0.44	43.93 ± 2.22	6.40 ± 0.81
	HFE (200 mg/kg)	17.55 ± 1.14	7.94 ± 0.10	14.33 ± 0.24	44.43 ± 0.44*	4.00 ± 0.55¥
	HFE (500 mg/kg)	15.50 ± 0.39	8.19 ± 0.21	15.45 ± 0.57	47.00 ± 0.35¥	2.20 ± 0.37¥

Sprague-Dawley rats were injected sub plantar with 0.1 ml of IFA or CFA into the right hind paw. The drug vehicle, dexamethasone (0.3 mg/kg) and HFE (50, 200, 500 mg/kg) were administered orally 1 h before arthritis induction and thereafter until the 28th day in the prophylactic approach and started on the 14th day after the induction of arthritis and daily till the 28th day in the therapeutic approach. Blood was collected from the tail vein on day 28 and a full blood count was done. Erythrocyte sedimentation rate (ESR) was determined using the standard Westergren method. Data presented show mean ± S.E.M for *n* ≥ 5. * (*P* ≤ 0.05), ** (*P* ≤ 0.01), # (*P* ≤ 0.001), ¥ (*P* ≤ 0.0001) values significantly different when compared with CFA arthritic control

### Prophylactic effect of *Holarrhena floribunda* on serum IL-1α, IL-6 and IL-10 levels

**Fig. 3:**
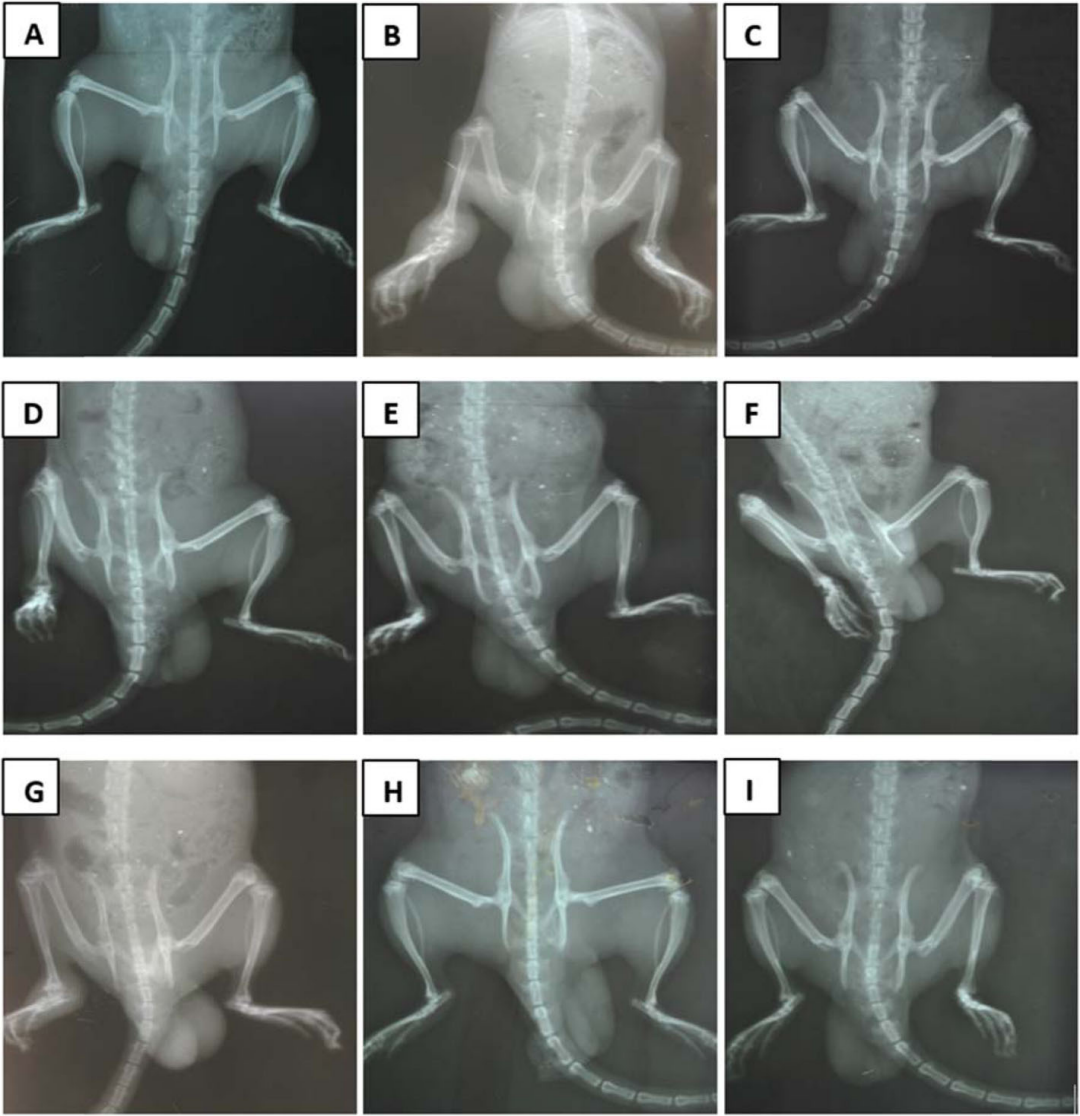
Effect of treatment on serum levels of IL-1α, IL-6 and IL-10 in control and adjuvant-induced arthritic rats. Sprague-Dawley rats were injected sub plantar with 0.1 ml of IFA or CFA into the right hind paw. The drug vehicle, dexamethasone (0.3 mg/kg) and HFE (50, 200, 500 mg/kg) were administered orally 1 h before arthritis induction and daily thereafter until the 28th day. IL-1α (A), IL-6 (B) and IL-10 (C) were assayed by ELISA. Data are presented as mean ± SEM for n ≥ 5. * (*P* ≤ 0.05), *** (*P* ≤ 0.001) values significantly different when compared with CFA arthritic control group

Serum IL-1α in the CFA control was 104.05 ± 27.46 pg/ml, compared to 5.23 ± 0.59 pg/ml in the IFA control group (Fig. 3 A). Treatment with HFE or dexamethasone resulted in reduced (*P* ≤ 0.0197) serum levels of the marker relative to the CFA control. Dexamethasone recorded IL-1α serum concentration of 21.53 pg/ml, which was not significantly different (*P* ≥ 0.5504) values for HFE.

Serum IL-6 increased from 0.45 ± 0.04 pg/ml in the IFA non-arthritic control group to 3.72 ± 0.30 pg/ml in the CFA arthritic control (Fig. 3 B). Treatment with HFE led to reductions in serum IL-6 concentration, which was significant (*P* = 0.0032) only for the 500 mg/kg dose. Dexamethasone reduced (*P* = 0.0149) serum IL-6, but the effect was not different (*P* ≥ 0.4348) from any of the HFE doses.

IL-10 concentration in serum from the CFA and IFA control groups were 25.17 ± 5.17 pg/ml and 657.66 ± 21.73 pg/ml, respectively (Fig. 3 C). Dexamethasone increased (*P* = 0.0003) serum IL-10. Similarly, HFE administration increased serum IL-10, but this was significant (*P* = 0.0462) only for the 500 mg/kg dose. The value for dexamethasone was not significantly different (*P* = 0.1079) from 500 mg/kg HFE.

### Effect of *Holarrhena floribunda* on enzymes and secondary messengers

**Fig. 4:**
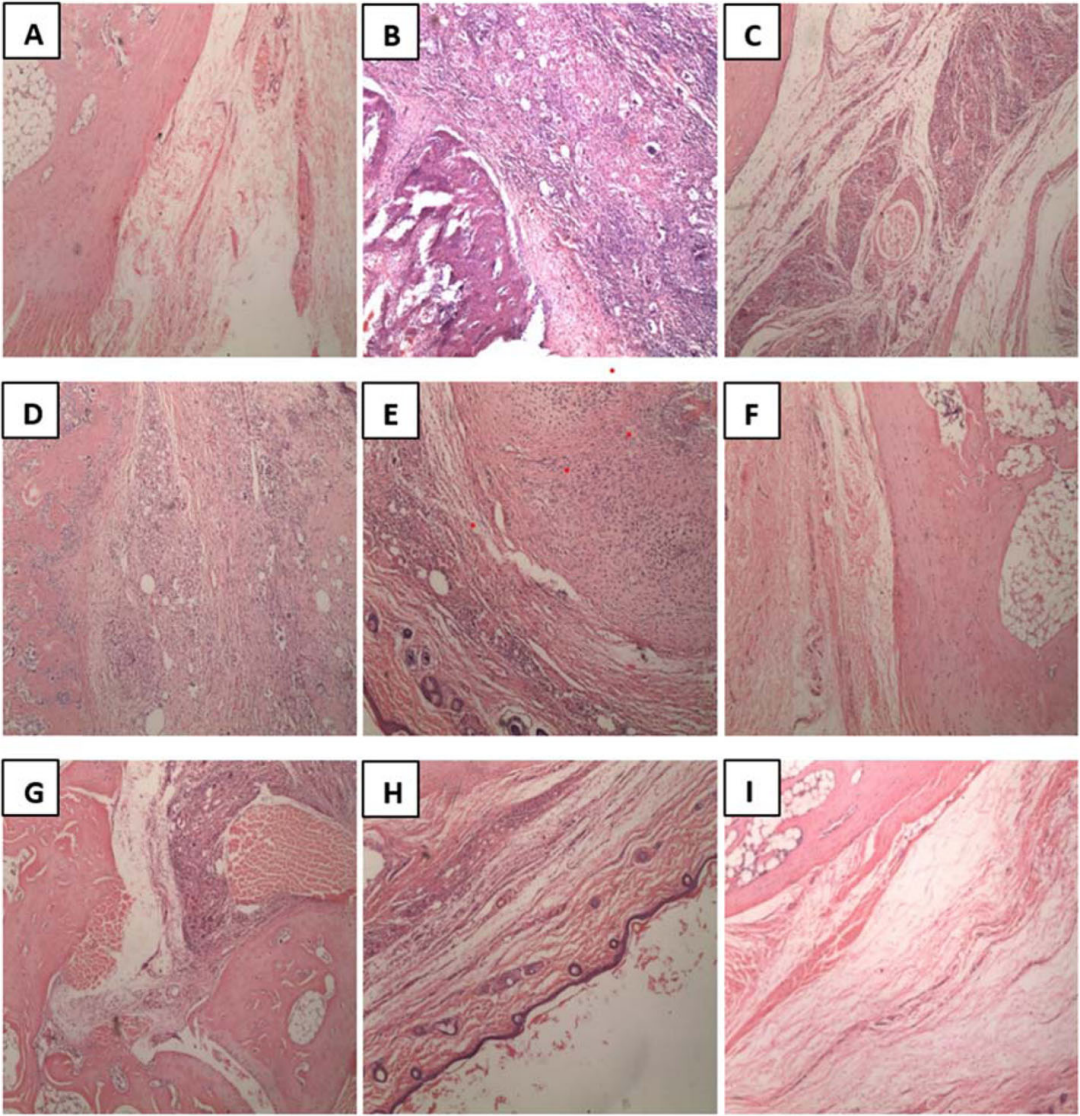
Effect of treatment on serum levels of PKA, PKC and cAMP in control and adjuvant-induced arthritic rats. Sprague-Dawley rats were injected sub plantar with 0.1 ml of IFA or CFA into the right hind paw. The drug vehicle, dexamethasone (0.3 mg/kg) and HFE (50, 200, 500 mg/kg) were administered orally 1 h before arthritis induction and daily thereafter until the 28th day. Serum PKA (A), PKC (B) and cAMP (C) were assayed by ELISA. Data are presented as mean ± SEM of n ≥ 5. * (*P* ≤ 0.05), ** (*P* ≤ 0.01), *** (*P* ≤ 0.001) values significantly different when compared with CFA arthritic control group

Arthritic CFA control animals showed a reduction in serum PKA, approximately three-fold from 25.41 ± 2.07 ng/ml to 7.71 ± 0.64 ng/ml (Fig. 4 A). Administration of HFE at 200 or 500 mg/kg increased serum PKA (*P* ≤ 0.0393). The value for dexamethasone (17.7 ng/ml) was not different (*P* ≥ 0.3779) from the values for the 200 and 500 mg/kg HFE doses.

Serum PKC was elevated at least four-fold, from 0.38 ± 0.04 ng/ml in the IFA non-arthritic control group to 1.78 ± 0.37 ng/ml in the untreated CFA control (Fig. 4 B). Both dexamethasone and 500 mg/kg HFE reduced serum PKC activity, to 38 and 69% of the CFA value, respectively. However, neither of the measured effects was significant (*P* ≥ 0.0958).

Induction of arthritis reduced serum cAMP from 16.53 ± 1.72 pg/ml in the IFA non-arthritic control to 4.95 ± 0.32 pg/ml in the CFA arthritic control (Fig. 4 C). Treatment with HFE increased (*P* ≥ 0.0938) serum cAMP levels compared to the CFA control group. The increase due to dexamethasone was also not significant (*P* = 0.4207).

## Discussion

The phytochemical profile of HFE includes phenolic compounds, alkaloids and saponins (see Additional file 1). Molecules from these three classes have variously been reported to exhibit anti-inflammatory bioactivities [23–26]. In this study, the inhibitory effect of *Holarrhena floribunda* stem bark hydro ethanol extract (HFE) on chronic inflammation was evaluated in Sprague-Dawley rats. The Complete Freund’s Adjuvant (CFA)-induced arthritis model was adopted because it has several characteristics, including serology, in common with human rheumatoid arthritis [27–29]. Adjuvant-induced arthritis is a T lymphocyte-dependent chronic inflammatory condition that develops in two phases: an acute, peri-articular inflammation phase followed by a chronic phase with joint and bone involvement [30, 31]. Both phases were observed in this study. Also, biological and immunological features of the immune response to CFA [27, 32], such as lesions or ulcers at the site of injection, peri-articular erythema, oedema, reduction of paw function and evidence of hyperalgesia were observed in arthritic control animals. The development of contralateral swelling at the CFA dose used in this study is consistent with previous observations [33–35]. Alleviation of the listed features were assessed in drug/extract-treated animals.

HFE showed its strongest anti-arthritic activity at the 500 mg/kg dose, at least similar to dexamethasone in both the prophylactic and therapeutic treatment models (Figs. 1 & 2; Table 1). Administration of HFE inhibited the development of paw oedema in the acute inflammation phase (Fig. 1; Plate 1; Tables 1 & 2) and suppressed development of polyarthritis in the chronic phase (Figs. 1 & 2; Plate 1, 2 and 3). Short-term inflammatory responses, as seen in the acute inflammation phase, are typically mediated by histamine via the H1 receptor [36, 37]. In a previous report, we have demonstrated the strong antihistaminic activity of HFE [22]. We acknowledge that data on the role of histamine in arthritis progression have traditionally been contradictory [38]. However, more recent reports and analyses suggest strong links, depending on histamine receptor type expression, between histamine and various hypersensitivity response mediators, particularly leukocytes and T cells [39] which are involved in the development of CFA-induced arthritis. It is our opinion that the contribution of histamine, via the H1 receptor, to the acute oedema following CFA administration in the adjuvant-induced arthritis model cannot be overlooked [37, 40, 41]. More relevant to the chronic inflammation phase and human rheumatoid arthritis, we point to the role of histamine receptors, particularly the H4 receptor, in affecting levels of both pro- and anti-inflammatory cytokines by T cell modulation [39, 42, 43]. The ligand-bound H4 receptor is reported to play both autocrine and paracrine roles [44] in the progression of long-term inflammatory responses, by mediating (1) MAPK activation; (2) enhanced Ca^2+^ release; (3) mast cell activation for expression of pro-inflammatory cytokines [45–48]; and (4) decrease in levels of anti-inflammatory cytokines [42].

We have previously suggested that HFE may be useful against COVID-19-associated inflammation [22]. Histamine involvement in COVID-19 progression has been suggested and/or described in several reports [49–52]. The anti-histaminic potential of HFE, again demonstrated in this study by HFE inhibition of both acute and chronic oedema due to CFA, could play a role in modulating the dysregulated immune response associated with COVID-19.

Another feature of chronic inflammation pathologies is contribution of the vasculature to the disease process by formation of hyperpermeable vessels with poor blood flow allowing the leakage of blood components and contributing to oedema [53]. Such vascular proliferation is also seen in CFA-induced inflammation [54]. The ability of HFE to reduce this pathology is suggested by the observed reduction in erythema and oedema relative to the arthritic control animals (Plate 1) [55–61]. The anti-arthritic effect of HFE was further demonstrated by inhibition of necrosis, vascular proliferation and bone loss at the joints (Plates 2 & 3; Table 1) [54, 62–64], leading to preservation of joint integrity [65, 66]. HFE evidently limited osteoclast differentiation and activity, shown by reduction in the extent of bone resorption (Plate 2). This inhibitory effect is indicative of interaction between HFE and factors including receptor activator of nuclear factor kappa B (NF-κB) ligand (RANKL), tumour necrosis factor-α (TNF-α), and prostaglandin E_2_ (PGE_2_), all of which promote osteoclast differentiation and action leading to bone resorption [65, 66].

Several lines of evidence point to pro-inflammatory cytokines, including IL-1, IL-2, IL-6 and TNFα, as major players in the onset and progression of adjuvant-induced arthritis [67–70]. Indeed IL-1 and TNFα have been reported to be responsible for the initiation and propagating of bone erosion and cartilage destruction [71–74]. An imbalance between the proinflammatory cytokines and anti-inflammatory cytokines, such as IL-10 and IL-4, precipitates inflammatory disease conditions, including rheumatoid arthritis [75, 76]. In this study, HFE suppressed serum levels of IL-1α and IL-6, but enhanced IL-10 (Fig. 3). IL-10 is actively involved in limiting immune-mediated inflammation resulting from infection, allergy, and autoimmunity [77, 78] Downstream effects of IL-10 include activation of Treg cells which supress T helper 17 cell responses, leading to inhibition of both pro-inflammatory cytokine production and autoimmunity [79–82]. The ability of HFE to suppress the pro-inflammatory IL-6 and increase serum IL-10 is suggestive of immunomodulatory potential.

Serum PKC activity was reduced (Fig. 4 A), possibly due to the potentially inhibitory effect of HFE on Ca^2+^ release due to the antihistaminic activity. PKC is activated by calcium ion and has been shown to be involved in propagation of the immune inflammatory response [83–86]. In contrast, HFE increased serum levels of PKA and cAMP (Fig. 4 B & C). cAMP and its effector, PKA, are implicated in the resolution of acute inflammation [87] and are generally regarded as anti-inflammatory [88–90]. Further, cAMP has been shown to inhibit histamine release from human mast cells [91] and is reported to be key in several endogenous processes involved in preventing acute inflammation from progressing to deleterious chronic inflammation [92]. HFE reduced WBC and ESR (Table 3), both of which are markers of inflammation status [55–61], compared with the CFA arthritic control. Notwithstanding comparable levels of inflammation reduction, however, WBC counts in HFE treatment groups were ≥ 199.6% (*P* ≤ 0.0013) that of dexamethasone. This observation is of particular interest, for example, with respect to patients who may require discontinuation of corticosteroid use due to excessive or undesirable generalised immunosuppression. Taken together, the data strongly support that HFE has anti-oedematogenic, anti-histaminic, anti-inflammatory, and immunomodulatory activities. The potential for use of HFE as a prophylactic agent is another key advantage over conventional anti-inflammatory agents, including the glucocorticoids routinely used in the treatment of rheumatoid arthritis.

## Conclusion

In both the prophylactic and therapeutic models used in this study, HFE has shown potent anti-inflammatory and immunomodulatory activities in Freund adjuvant-induced arthritic Sprague Dawley rats. HFE indicated better prophylactic than therapeutic effect, modulating serum levels of histamine, IL-1α, IL-6, IL-10, cAMP and protein kinases A and C. Our data provide unequivocal evidence of the potential of HFE for use in management of rheumatoid arthritis and similar chronic inflammatory conditions. Further study is underway to elucidate the mechanism(s) of action.

## Materials and methods

### Preparation of extract

*Holarrhena floribunda* (G.Don) T. Durand & Schinz stem bark was collected from Kwahu-Asakraka (6°38′02.6 N″;0°41′37.5″W), Eastern Region, Ghana. The plant name has been checked with http://www.theplantlist.org on 28th Nov. 2020. The plant material was identified and authenticated by the Plant Development Department of Centre for Plant Medicine Research (CPMR), Mampong-Akuapem, Ghana. The Plant Development Department has a licence from the Forest Services Division of Forestry Commission of Ghana to source for plant material from the arboretum of CPMR and the wild. A voucher specimen (no. 05/13) is deposited at the herbarium of CPMR. The material was washed thoroughly, air-dried and milled into coarse powder. Powdered stem bark (1 kg) was macerated in 5 L of 70% v/v ethanol with periodic stirring, decanted after 72 h and filtered through Whatman no 1 filter paper. Ethanol was evaporated (BUCHI Rotavap; Flawil, Switzerland). The aqueous concentrate was lyophilised, stored at 4 °C and was reconstituted in sterilised distilled water at doses of 50, 200 and 500 mg/kg for use as *H. floribunda* bark hydro ethanol extract (HFE). The doses were selected based on anecdotal ethnobotanical information which suggested a minimal therapeutic dose of 50 mg/kg, and data from preliminary studies in our laboratory to determine effective doses for HFE anti-inflammatory activity [22].

### Experimental animals

All animal experiments were approved by the Ethics Committee of CPMR (approval number CPMR/M.6-PT3/2018). Male Sprague Dawley rats (200–220 g) were housed in the Animal Experimentation Unit of CPMR under ambient laboratory conditions: temperature 26 ± 2 °C, relative humidity 60–70%, normal light/dark cycle of 12 h each). Animals were randomly assigned to groups labelled either as control (vehicle or positive) or extract treatment groups. All animals were acclimatized for 7 days in the designated experimentation room before the start of experiments. The animals were trained to allow cooperation with restraint and other handling procedures. Throughout the period, the animals were handled in accordance with internationally accepted principles of laboratory animal use and care (EEC Directive 2010/63/EU). The animals were allowed access ad libitum to pelleted feed (Agricare Ghana Ltd) and sterilized drinking water. The wellbeing of animals used in all experimental procedures was continuously monitored at most at 12-h intervals. Death of an animal was not used as the endpoint in any experiment. Animals found to be visibly morbid were euthanised to alleviate pain and distress. Criteria for euthanasia included body temperature below 34 °C, laboured respiration, reduced exploration, reduced grooming, inability to access food and water, and lack of response to manipulation [93, 94]. Euthanasia was by pentobarbital sodium *i.p.*, 800 mg/kg [95].

### Induction of arthritis

Complete Freund’s adjuvant (CFA) was constituted as a 5 mg/ml suspension of heat-killed *Mycobacterium tuberculosis* triturated in sterile paraffin oil (KAMA Pharmaceutical Industries, Ghana) [96]. Arthritis was induced in the Sprague-Dawley rats (SDRs) by a one-time sub-plantar injection of the right hind paw with 100 μl CFA. Non-arthritic control animals received 100 μl of sterile paraffin oil (KAMA Pharm, Ghana), subsequently referred to as Incomplete Freund’s adjuvant (IFA). A plethysmometer (Ugo Basile, Italy) was used to measure paw volume for both the injected and contralateral hind paws before injection of CFA or IFA, and then every other day after that for 28 days [97]. The oedema component of inflammation was computed as the percent change in paw volume from day zero at each time point.

### Administration of Extract

Each treatment group had six rats. Dexamethasone (Sigma-Aldrich, St Louis) was used as the positive control agent. Preparations for treatment (dexamethasone or HFE) were freshly constituted and administered daily by oral gavage. For the prophylactic drug protocol, dexamethasone (0.3 mg/kg b.w.) or HFE (50, 200 or 500 mg/kg b.w.) was administered 1 h before induction of arthritis [98]. In the therapeutic protocol, treatment started on day 14 after induction of arthritis. In both cases, treatment was terminated on day 28.

### Assessment of anti-arthritic effect

Protective or curative properties of HFE were assessed using four indices of arthritic damage: (1) maximal and total oedema, (2) arthritis score from photographs and X-ray images, (3) histopathology and (4) haematology.

#### Maximal and total oedema

Maximal and total oedema responses were compared between drug-treated groups and untreated control groups. To obtain oedema responses, the foot volumes were individually normalized as percentage of change from the value at day zero, and then averaged for each treatment group. Mean percent change in paw volume for each treatment was calculated as:

$$ \% Change\ in\  paw\  volume=\left[\frac{P{V}_t-P{V}_0}{P{V}_0}\right]\times 100 $$

Where *PV*_*0*_ and *PV*_*t*_ are respectively the paw volumes at times *0* and timepoint *t*, respectively.

Total oedema induced was determined as area under the time course curves (AUC). Percent inhibition of total oedema for each treatment was calculated as:

$$ \% Inhibition\ of\ oedema=\left[\frac{AU{C}_{control}- AU{C}_{treated}}{AU{C}_{control}\ }\right]\times 100 $$

#### Arthritis score

Severity of arthritis on day 28 was represented by arthritis scores for the injected and contralateral hind paws, assessed by photography and radiography [99]. The extent of oedema was scored blindly from photographs (FE-5050, OLYMPUS, Tokyo, Japan) on a scale of 0–4 interpreted as 0 = un-injected paw with no swelling; 1 = slight swelling and/or erythema; 2 = low to moderate oedema and/or erythema; 3 = pronounced oedema and/or erythema with limited joint use; 4 = excess oedema and/or erythema with joint rigidity.

X-ray images were taken on industrial X-ray film (Fuji Photo Film, Tokyo, Japan) using an X-ray machine (Philips, Eindhoven, Netherlands) operated at 52 kV against 3.2 mA s^− 1^ with a tube-to-film distance of 110 cm for lateral projection. Severity of joint and bone deformation was blindly scored according to the extent of osteoporosis, joint spaces and joint structure [100] on a scale of 0–4, interpreted as 0 = no degenerative joint changes; 1 = slight soft tissue volume, joint space, subchondral erosion, periostitis, osteolysis, subluxation or degenerative joint changes; 2 = low to moderate soft tissue volume, joint space, subchondral erosion, periostitis, osteolysis, subluxation or degenerative joint changes; 3 = pronounced soft tissue volume, joint space, subchondral erosion, periostitis, osteolysis, subluxation or degenerative joint changes; 4 = excess soft tissue volume, joint space, subchondral erosion, periostitis, osteolysis, subluxation or degenerative joint changes.

#### Histology

Rats were euthanized by cervical dislocation on day 28. The injected paws were amputated above the ankle and fixed in 4% formalin. Hair on the paws were trimmed and the paws were placed in decalcifying solution (14% EDTA) for 10 days. Decalcified paws were embedded in paraffin, sectioned at 4 μm, stained with haematoxylin and eosin, and observed under a light microscope (100 × magnification; Dialux 22; Leitz, Wetzlar, Germany). Changes in joint bone tissue were scored blindly on a scale of 0–3, interpreted as 0 = absence of synovial hyperplasia, pannus, bone erosion and inflammatory cells; 1 = mild presence of synovial hyperplasia, pannus, bone erosion, and presence of inflammatory cells; 2 = moderate presence of synovial hyperplasia, pannus, bone erosion, and presence of inflammatory cells; 3 = severe presence of synovial hyperplasia, pannus, bone erosion, and presence of inflammatory cells.

#### Haematology

Blood was collected on day 28 for both prophylactic and therapeutic treatments by tail bleeding into EDTA tubes (Cland Medical Instruments, Zhejiang, China) for haematological analysis (Abacus 380, Budapest, Hungary) or estimation of erythrocyte sedimentation rate (Curtin Matheson Scientific, Houston, TX, respectively.

### Assessment of serum indicators: IL-1α, IL-6, IL-10, PKA, PKC, cAMP

Blood collected from the tail vein into vacutainer gel and clot activator tubes (SG Biotech, Middlesex, England) on day 28 were left to clot at room temperature and centrifuged at 1000×g for 10 min to obtain serum obtained. Serum levels of interleukin 1-alpha, interleukin 6, interleukin 10 (Abcam Plc, Cambridge, UK), protein kinases A and C, and cyclic adenosine monophosphate (MyBioSource, San Diego, CA) were each measured in triplicate with the appropriate rat ELISA kit according to the manufacturer’s guidelines.

### Statistical analyses

All graphing and analyses were performed with GraphPad Prism for Windows Version 8 (GraphPad, San Diego, CA). Data were analysed by one-way or two-way (treatment x time) repeated measure analysis of variance followed by Tukey’s multiple comparisons test. Indications of significance are reported as multiplicity adjusted *P* values. Unless otherwise stated, all comparisons described are relative to the CFA arthritic control group. Differences in means were considered statistically significant at *P* ≤ 0.05.

## Supplementary Information

**Additional file 1 **. Phytochemical constituents of *Holarrhena floribunda* stem bark hydro ethanol extract. Qualitative phytochemical composition of *Holarrhena floribunda* stem bark hydro ethanol extract was determined using the methods described by Sofowora (1993) and Trease and Evans (2002).

## Data Availability

The datasets used and/or analysed during the current study are available from the corresponding author on reasonable request.

## Abbreviations

cAMP: Cyclic AMP
CFA: Complete Freund’s adjuvant
COVID-19: Corona virus disease 2019
ESR: Erythrocyte sedimentation rate
IFA: Incomplete Freund’s adjuvant
IL: Interleukin
MAPK: Mitogen-activated protein kinase
PKA: Protein kinase A
PKC: Protein kinase C
PGE2: Prostaglandin E2
RANKL: Receptor activator of nuclear factor kappa B (NF-κB) ligand
TNFα: Tumor necrosis factor alpha
