# Supplementary material for: Prophylactic or therapeutic administration of Holarrhena floribunda hydro ethanol extract suppresses complete Freund’s adjuvant-induced arthritis in Sprague-Dawley rats
Source: J Inflamm (Lond). 2022 Mar 5;19:3. doi: 10.1186/s12950-022-00301-2 (PMC8897772; doi:10.1186/s12950-022-00301-2)
Supplement: Supplementary file 1 — Additional file 1 . Phytochemical constituents of Holarrhena floribunda stem bark hydro ethanol extract. Qualitative phytochemical composition of Holarrhena floribunda stem bark hydro ethanol extract was determined using the methods described by Sofowora (1993) and Trease and Evans (2002). [file 12950_2022_301_MOESM1_ESM.docx]

**Additional File 1**

**Phytochemical constituents of *Holarrhena floribunda* stem bark hydro ethanol extract**

| Phytochemical | Result |
| --- | --- |
| Saponins | Present |
| Reducing sugar | Present |
| Triterpenes | Absent |
| Phytosterols | Absent |
| Flavonoids | Absent |
| Phenolic compounds | Present |
| Polyuronides | Absent |
| Alkaloids | Present |
| Cyanogenic glycosides | Absent |

Qualitative phytochemical composition of *Holarrhena* *floribunda* stem bark hydro ethanol extract was determined using the methods described by Sofowora (1993) and Trease and Evans (2002).
